# Overexpression of microRNA-129-5p in glioblastoma inhibits cell proliferation, migration, and colony-forming ability by targeting *ZFP36L1*

**DOI:** 10.17305/bjbms.2019.4503

**Published:** 2020-11

**Authors:** Xu Guo, Haozhe Piao, Ye Zhang, Peixin Sun, Bing Yao

**Affiliations:** Department of Neurosurgery, Cancer Hospital of China Medical University, Liaoning Cancer Hospital and Institute, Shenyang, China

**Keywords:** Glioblastoma multiforme, GBM, microRNA, miR-129-5p, ZFP36L1, migration, proliferation, colony-forming ability, tumor-suppressor

## Abstract

Glioblastoma multiforme (GBM) is a highly invasive cancer with a high recurrence rate. The prognosis of GBM patients remains poor, even after standard surgical resection combined with chemoradiotherapy. Thus, there is an urgent need for new therapeutic targets in GBM. In recent years, microRNAs have received considerable attention due to their important role in tumor development and progression. In this study, we investigated the role of miR-129-5p and miR-129-5p/ZFP36L1 axis in GBM tumorigenesis. Analysis of GSE103228 microarray data from the GEO database showed that miR-129-5p was significantly downregulated in GBM vs. normal brain tissues. Quantitative reverse transcription PCR analysis of miR-129-5p expression in seven GBM cell lines (LN229, A172, U87, T98G, U251, H4, and LN118) vs. normal human astrocytes (NHA) showed miR-129-5p was significantly downregulated in GBM cells. Overexpression of miR-129-5p in LN229 and A172 cells significantly suppressed cell proliferation, migration, invasion, and colony-forming ability. Target Scan analysis identified *ZFP36L1* as the target of miR-129-5p. UALCAN dataset analysis found that *ZFP36L1* was significantly upregulated in GBM vs. normal brain tissues, and high *ZFP36L1* expression was positively associated with poor survival of GBM patients. Western blot analysis demonstrated that ZFP36L1 was significantly upregulated in seven GBM cell lines vs. NHA. Overexpression of miR-129-5p in LN229 and A172 cells significantly inhibited ZFP36L1 mRNA and protein expression, while overexpression of ZFP36L1 in LN229 and A172 cells reversed miR-129-5p-mediated inhibition on GBM tumorigenesis. Our results revealed an important role of miR-129-5p in the negative regulation of *ZFP36L1* expression in GBM, suggesting new candidates for targeted therapy in GBM patients.

## INTRODUCTION

Glioma accounts for the largest category of primary brain tumors among which glioblastoma multiforme (GBM) is the most aggressive brain tumor. GBM accounts for about 14.7% of all primary and metastatic tumors of the brain [1]. The incidence of GBM has been increasing year by year, while the incidence of other glioma types has been gradually decreasing [2]. In the USA and Europe, the incidence of GBM is 2 to 3 per 100,000 people [3]. GBM is the most malignant type of astrocytoma, and the clinical manifestations are headache, convulsions, and psychiatric symptoms. Other typical features of GBM are fast growth and high malignancy. Although surgery combined with radiotherapy and chemotherapy has been widely used for the treatment of GBM patients, the prognosis is still very poor with a median survival of about 12 months, a 2-year survival rate of only 26–33%, and a 5-year survival rate of about 4–5% [2,4]. In order to provide a better treatment strategy for GBM patients, it is necessary to identify potential therapeutic targets for GBM and to clarify the underlying mechanism.

MicroRNAs are a class of endogenous non-coding single stranded RNAs of ~22 nucleotides in length, which participate in post-transcriptional regulation of gene expression in both plants and animals [5]. Studies have demonstrated that microRNAs play a crucial regulatory role in the development, differentiation, and apoptosis of normal cells, as well as an oncogenic or tumor suppressive role in cancer formation and progression [6,7]. At the molecular level, microRNAs target the 5’- or 3’-untranslated region (UTR) of the target genes and subsequently regulate cellular functions, such as tumor proliferation, migration, and invasion. Abnormal expression of microRNAs was found in chronic lymphocytic leukemia [8], breast cancer [9], non-small cell lung cancer [10], pancreatic cancer [11], and liver cancer [12]. Recent studies showed that microRNAs are also involved in glioma development [13-17]. Overexpression of miR-148a and miR-378-3p suppressed GBM cell proliferation, migration, and invasion by targeting integrin alpha 9 (ITGA9) and transmembrane 4 superfamily member 17 (tetraspanin 17, Tspan17), respectively [18,19]. In addition, the expression of microRNAs was found to be associated with chemoresistance of glioblastoma and non-small cell lung cancer [20-22]. Upregulation of miR-145 enhanced the sensitivity of GBM stem cells to demethoxycurcumin, and overexpression of miR-29a significantly inhibited the proliferation of GBM stem cells.

In recent years, miR-129-5p has received considerable attention as a driver of Alzheimer’s disease [23], osteogenesis [24], and intervertebral disk degeneration [25]. Furthermore, miR-129-5p was reported to be involved in the tumorigenesis of gastric [26-28], pancreatic [29], laryngeal [30], prostate [31], and lung cancer [32] as well as of hepatocellular carcinoma [33]. Recent reports demonstrated that miR-129-5p was able to regulate the proliferation and invasion of GBM cells lines [16,34]. However, despite these studies, the specific regulatory mechanisms of miR-129-5p in glioma remain unclear and require further investigation.

ZFP36 ring finger protein-like 1 (ZFP36L1) is a member of the ZFP36 family of RNA-binding proteins [35], which contains two standard repeating zinc finger motifs (CCCH). The CCCH motif binds to the AU-rich elements (AREs) in the 3’-UTR of the target mRNA resulting in mRNA instability [36]. It was reported that ZFP36L1 was functionally involved in post-transcriptional regulation of bile acid metabolism in hepatocytes, myogenesis, and lymphocyte development [37-40]. Disruption of ZFP36L1 expression in mice led to death on embryonic day 11 [41]. Increased expression of ZFP36L1 inhibited cell growth in lung and colorectal cancer cell lines [42,43]. However, little is known about the biological role of ZFP36L1 and its regulatory factors (such as microRNAs) in GBM tumorigenesis.

In this study, we investigated the role of miR-129-5p and miR-129-5p/ZFP36L1 axis in GBM tumorigenesis. First, we used the GSE103228 microarray data from the gene expression omnibus (GEO) database to analyze differentially expressed microRNAs between GBM and normal tissues and found that miR-129-5p was significantly downregulated in GBM tissues. Further, our UALCAN dataset analysis showed that ZFP36L1 was significantly upregulated in GBM vs. normal brain tissues. Quantitative reverse transcription PCR (qRT-PCR) analysis of miR-129-5p expression in seven GBM cell lines vs. normal human astrocytes (NHA) showed miR-129-5p was significantly downregulated in GBM cells. Subsequently, we confirmed that overexpression of miR-129-5p inhibited the proliferation, migration, invasion, and colony-forming ability of LN229 and A172 GBM cells. Furthermore, we identified that ZFP36L1 was the downstream target gene of miR-129-5p. Overexpression of miR-129-5p in LN229 and A172 cells inhibited the expression of ZFP36L1, while forced expression of ZFP36L1 reversed miR-129-5p-mediated inhibition on GBM tumorigenesis, including tumor proliferation, migration, and colony-forming ability. Our results reveal an important role of the miR-129-5p/ZFP36L1 axis in GBM tumorigenesis and shed a new light on targeted therapy in GBM.

## MATERIALS AND METHODS

### Analysis of microarray data

The microRNA and mRNA expression profile GSE103228 was selected from the GEO database (https://www.ncbi.nlm.nih.gov/geo/). The profile included mRNA and microRNA expression of five human GBM and five normal brain tissues. Analysis of microRNA expression was performed on the GPL18058 platform (Exiqon miRCURY LNA microRNA array, 7th generation). The differentially expressed microRNAs in GBM samples were further analyzed by the GEO’s R language analysis tool (http://www.ncbi.nlm.nih.gov/geo/geo2r/). Adjusted *p* values were applied based on the Benjamini-Hochberg method to minimize the false discovery rate. To determine dysregulated microRNAs in GBM samples, the cut-off values were defined as the fold change >2 and adjusted *p* < 0.05 was used. After removing unknown microRNAs and missing information, we found that miR-129-5p was among the top three dysregulated microRNAs and was selected for further analysis.

### Identification of microRNA target genes

The candidate target genes of miR-129-5p were identified using TargetScan (http://www.targetscan.org/vert_72/), which is dedicated to the analysis of mammalian microRNA target genes. Based on the cumulative weighted context++ score, ZFP36L1 was among the top 25 target candidates for miR-129-5p. After literature search, pre-analysis of TCGA, and removal of proteins with unknown function, ZFP36L1 remained as the promising candidate gene and was selected for further analysis.

### UALCAN dataset analysis

The UALCAN (http://ualcan.path.uab.edu/index.html) database provides an easy access to publicly available cancer transcriptomics data (namely, TCGA and MET500 transcriptome sequencing). In this study, UALCAN was used to identify biomarkers and to validate *in silico* potential genes of interest. In addition, *ZFP36L1* gene expression and patient survival information based on the gene expression were analyzed with the UALCAN dataset to explore ZFP36L1 impact on the prognosis of patients with GBM.

### Cell culture

Normal human astrocyte (NHA) cells and human GBM cell lines (LN229, A172, U87, T98G, U251, H4, and H118) were obtained from the Institute of Biochemistry and Cell Biology, Chinese Academy of Sciences (Shanghai, China). These cells were cultured in RPMI-1640 medium (Invitrogen, Carlsbad, CA, USA) containing 10% fetal bovine serum (FBS, Invitrogen) in a humidified atmosphere of 5% CO_2_ at 37°C.

### RNA isolation and qRT-PCR

Total RNAs were extracted from cultured cells as described previously [44]. In brief, total RNAs were extracted by TRIZOL reagent (Gibco Invitrogen), and the quality and integrity of the extracted RNAs were determined by agarose gel electrophoresis. Poly(A) tails were added according to the protocol of the Poly (A) Tailing Kit (Tiangen, Beijing, China). Complementary DNA (cDNA) was synthesized using the PrimeScriptTM RT reagent Kit with gDNA Eraser (Takara, Dalian, China) and gene-specific primers or random primers. qRT-PCR was performed on a LightCycler 480 II Real-Time PCR system (Roche Diagnostics, Basel, Switzerland) using SYBR^®^ Green (Takara). Glyceraldehyde 3-phosphate dehydrogenase (GAPDH) and U6 small nuclear RNA (snRNA) were employed as endogenous controls for mRNA and microRNA, respectively. The comparative Ct method was used to calculate the relative expression of RNA. The primers used in this study were as follows:

miR-129-5p:5’-CGCTACCCTGTAGATCCGAATTTGTG-3’; U6: 5’-CGCTTCGGCAGCACATATAC-3’ (forward) and 5’-TTCACGAATTTGCGTGTCAT-3’ (reverse); ZFP36L1: 5’-ACCACCACCCTCGTGTCTG-3’ (forward) and 5’-TGCCCACTGCCTTTCTGT-3’ (reverse); GAPDH: 5’-CGGATTTGGTCGTATTGGG-3’ (forward) and 5’- CTGGAAGATGGTGATGGGATT-3’ (reverse).

### Western blot analysis

Western blot analysis was performed as described previously [45]. In brief, cells were lysed with lysis buffer (Beyotime, Shanghai, China) containing 1 mmol/L phenylmethylsulfonyl fluoride (PMSF). After centrifugation at 14,000 × g for 10 min at 4°C, the extracted proteins were resuspended, and the concentrations were measured using the bicinchoninic acid (BCA) protein assay kit (Beyotime). Equal amounts (30 µg) of protein were resolved by SDS-PAGE and electro-transferred onto the PVDF membrane (Millipore). The immunoblots were then blocked with 5% fat-free milk for 1 h, and further incubated overnight with primary antibodies against ZFP36L1 (1:1000 dilution, ab42473, Abcam, USA) and GAPDH (1:3000 dilution, ab181602, Abcam, USA) at 4°C. After incubation with peroxidase-conjugated secondary antibodies for 1 h, the blots were washed and then visualized using an electrochemiluminescence (ECL) detection kit (ThermoBiotech Inc, Rockford, IL, USA). The chemiluminescent signal on the blots was determined and quantified using densitometric software (Bio-Rad, California, USA).

### Transfection

Transfections were performed using the Lipofectamine 3000 Reagent (Invitrogen) according to the manufacturer’s instruction. For the transfection of microRNAs, 50 nmol/L of miR-129-5p mimics (5’-CUUUUUGCGGUCUGGGCUUGC-3’) and/or negative control miR-NC (5’-UUGUACUACACAAAAGUACUG-3’) were added to each transfection well (6-well culture plate) containing 2 mL culture medium. After 48 h of transfection, total RNAs and proteins were collected for further analysis. For the overexpression of ZFP36L1, 2 × 10^3^ of LN229 and A172 cells were seeded onto 6-well plates. After overnight culture, the cells were transfected with 0.75 µg/mL of pcDNA3.1 (control) or pcDNA3.1-ZFP36L1 for 48 h. The transfection efficiency of ZFP36L1 was evaluated by qRT-PCR and Western blot analysis. In order to visualize and count the numbers of cells, cells were fixed with 4% paraformaldehyde at 4°C for 60 min. After washing twice with ice-cold PBS, cells were stained with impurity-free crystal violet for 2 min and imaged under a microscope.

### Cell proliferation assay

Cell proliferation was determined using the Cell Counting Kit-8 (CCK-8) kit (Dojindo, Japan) according to the manufacturer’s instructions. In brief, cells (3000 cells/well) were transfected with NC (transfection reagent control), miR-NC (miRNA sponge-negative scramble control), or miR-129-5p mimics and cultured in 96-well plates. After 0, 24, 48, and 72 h of incubation, 100 µL of culture supernatant from each well was collected and transferred to another 96-well plate and then incubated with 10 µL of CCK-8 solution at 37°C for 4 h. The absorbance was measured at 450 nm wavelength using spectrophotometry (BioTek, USA).

### Wound healing assay

Cells were seeded in 6-well plates and then treated with NC, miR-NC, or miR-129-5p mimics. Linear scratch wounds were created on cell monolayers using a sterile 200-µL pipette tip, and the scratched areas were imaged at ×100 magnification using a Leica DMI3000B computer-assisted microscope (Leica, Buffalo Grove, IL, USA). Images were captured at 0, 24, 48, and 72 h after scratching and analyzed using Image-Pro Plus v6.0 image analysis software (Media Cybernetics, Rockville, MD, USA). The remaining wound area was calculated to estimate the migration distance of the cells.

### Matrigel invasion assay

Matrigel invasion assay was performed using Transwell plates (BD, Biosciences, CA, USA). Transwell membrane inserts were precoated with Matrigel (BD), and the analyzed cells were appropriately seeded onto the upper compartment of the chamber. The bottom chamber contained complete RPMI-1640 medium, and the upper chamber contained serum-free medium. After 24 h of incubation at 37°C, the cells on the Matrigel-coated upper surface were wiped off with a cotton swab, and the invaded cells throughout the filter were fixed and stained with Giemsa at 37°C for 3 h. The images of invaded cells were photographed with a microscope (Leica) at × 100 magnification. For each membrane isolated from triplicate chambers, the number of invaded cells was counted in three randomly selected fields. The results were presented as bar graphs.

### Statistical analysis

All statistical analyses were performed using IBM SPSS Statistics for Windows, Version 20.0 (IBM Corp., Armonk, NY, USA). Data were expressed as mean ± SD. The χ^2^ test, Student’s *t*-test, and one-way ANOVA analysis were used for comparisons. A value of *p* < 0.05 was considered statistically significant.

## RESULTS

### Downregulated miR-129-5p expression in GBM tissues and cell lines

**FIGURE 1:**
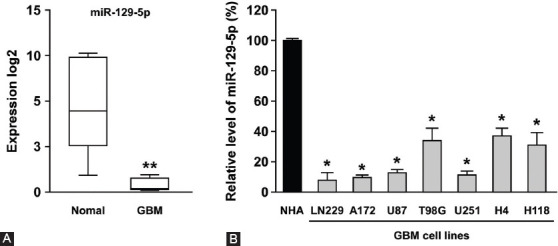
MiR-129-5p was downregulated in GBM tissues and cell lines. (A) The microarray dataset GSE103228 was used to examine whether miR129-5p expression was associated with GBM tumorigenesis. The expression level of miR-129-5p was significantly downregulated in five GBM tissues vs. five normal brain tissues. The expression values were calculated using log2 transformed RPM values. (B) MiR-129-5p was significantly downregulated in all GBM cell lines (LN229, A172, U87, T98G, U251, H4, and H118) vs. NHAs, with a reduction of approximately 60–90%. Differences were found to be statistically significant at *p < 0.05 and **p < 0.005. GBM: Glioblastoma multiforme; RPM: Reads per million; NHAs: Normal human astrocytes.

The microarray dataset GSE103228 was used to examine whether miR129-5p expression was associated with GBM tumorigenesis. After analyzing 3557 differentially expressed microRNAs, we found that the expression of miR-129-5p was significantly downregulated in GBM tissues vs. normal brain tissues (*p* < 0.005, Figure 1A). The decreased expression of miR-129-5p in GBM was further validated using NHAs and seven GBM cell lines, including LN229, A172, U87, T98G, U251, H4, and LN118. As shown in Figure 1B, miR-129-5p was significantly downregulated in all GBM cell lines with a reduction of approximately 60–90% compared to NHAs, suggesting that miR-129-5p may play an important role in the tumorigenesis of GBM.

### MiR-129-5p inhibited GBM tumorigenesis

**FIGURE 2:**
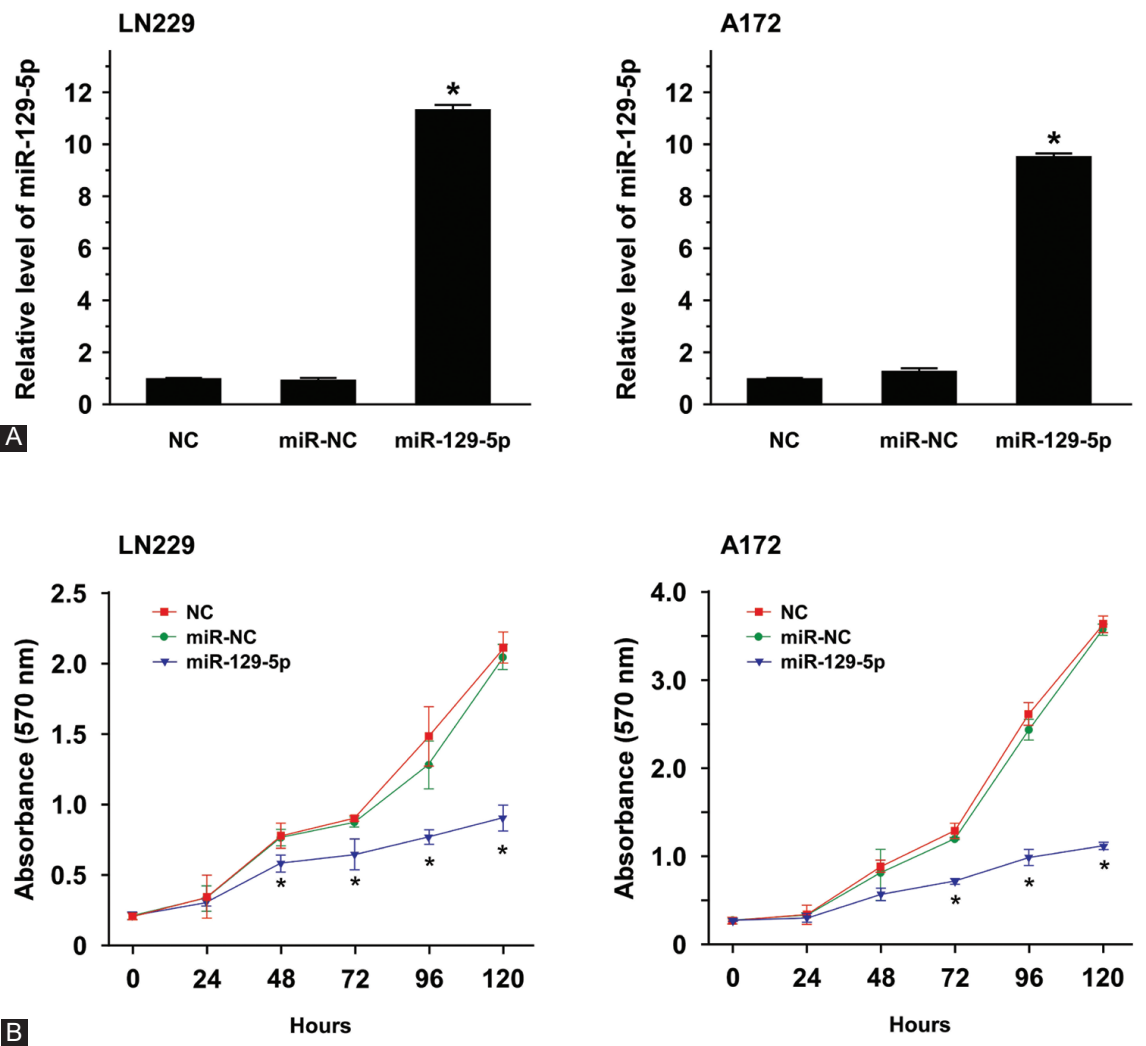
Overexpression of miR-129-5p reduced the proliferation ability of LN229 and A172 cells. (A) Quantitative reverse transcription PCR was used to determine the relative expression of miR-129-5p in LN229 and A172 cells transfected with NC (transfection reagent control), miR-NC (miRNA sponge-negative scramble control), or miR-129-5p mimics. Compared to miR-NC group, miR-129-5p level was significantly increased in miR-129-5p mimics group, to about 11.5-fold and 9.5-fold in LN229 and A172 cells, respectively. (B) The proliferation of LN229 and A172 cells was significantly inhibited by the overexpression of miR-129-5p after 72, 96, and 120 h of culture. The cell proliferation was detected by CCK-8 assay. Data are presented as the means of triplicate experiments. Differences were found to be statistically significant at *p < 0.05.

To explore the tumorigenic role of miR-129-5p in GBM, the two GBM cell lines LN229 and A172 with the least expression of miR-129-5p were selected. We first overexpressed miR-129-5p in LN229 and A172 cells and then examined whether miR-129-5p could affect GBM cell proliferation, migration, invasion, and colony-forming abilities. Compared to NC group, transfection with miR-NC did not alter the expression of miR-129-5p in LN229 and A172 cells (Figure 2A). Compared to miR-NC group, the level of miR-129-5p was significantly increased in miR-129-5p mimics groups, to about 11.5-fold and 9.5-fold in LN229 and A172 cells, respectively.

Next, CCK-8 assay was performed to examine whether miR-129-5p affects the cell proliferation ability. The overexpression of miR-129-5p significantly inhibited the proliferation of LN229 and A172 cells after 72, 96, and 120 h of culture (Figure 2B), indicating that miR-129-5p was functionally involved in the tumor cell proliferation.

**FIGURE 3:**
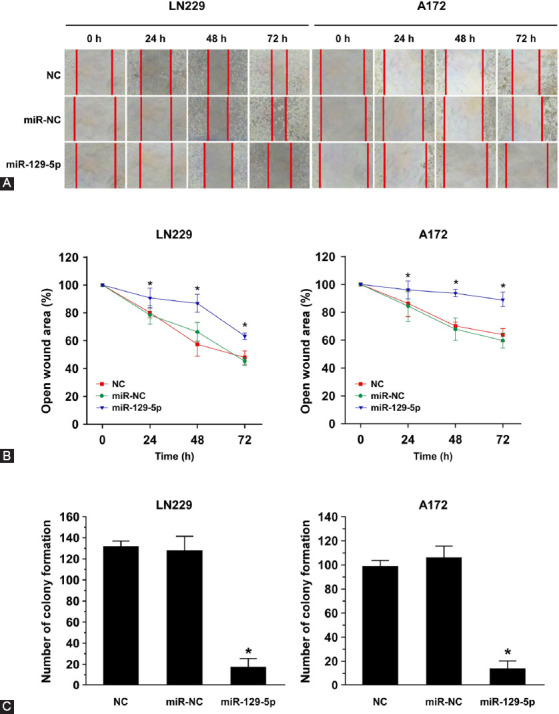
MiR-129-5p overexpression suppressed the migration ability of LN229 and A172 cells. (A) Photographs of artificially induced wounds in the monolayers of LN229 and A172 cells with miR-129-5p overexpression at different time points. NC (transfection reagent control) and miR-NC (miRNA sponge-negative scramble control) groups exhibited high motility, while the overexpression of miR-129-5p reduced the wound healing ability after 72 h of culture. (B) The wound-healing results were expressed as the percentage of migration ability at 0 h (100%). The wound closure rates of LN229 and A172 cells were significantly lower in miR-129-5p mimics compared with miR-NC group at 24, 48, and 72 h. Data are presented as the means of triplicate experiments. Differences were found to be statistically significant at *p < 0.05. (C) Overexpression of miR-129-5p markedly inhibited the colony-forming abilities of LN229 and A172 cells, i.e., it reduced the numbers of colony formation. Differences were found to be statistically significant between miR-129-5p and miR-NC at *p < 0.05.

Further, miR-129-5p-mediated proliferation and migration inhibition in GBM was examined by wound-healing assay, a classic method for a comprehensive analysis of cell proliferation and migration. As shown in Figure 3A, the two control groups (NC and miR-NC) of LN229 and A172 cells exhibited high motility, while the overexpression of miR-129-5p in mimics group reduced the wound healing ability after 72 h of culture. The wound closure rates of LN229 and A172 cells transfected with miR-129-5p mimics were significantly lower at 24, 48, and 72 h compared to those in miR-NC-transfected cells (*p* < 0.05; Figure 3B).

Using colony forming assay, we next examined whether miR-129-5p could affect the ability of GBM cells to form colonies. The results showed that the overexpression of miR-129-5p significantly inhibited colony formation in both LN229 and A172 cells (Figure 3C).

Taken together, the results of CCK-8, wound-healing, and colony-forming assay suggested that miR-129-5p can suppress GBM cell proliferation, migration, and colony-forming ability.

### ZFP36L1 as a target gene of miR-129-5p

**FIGURE 4:**
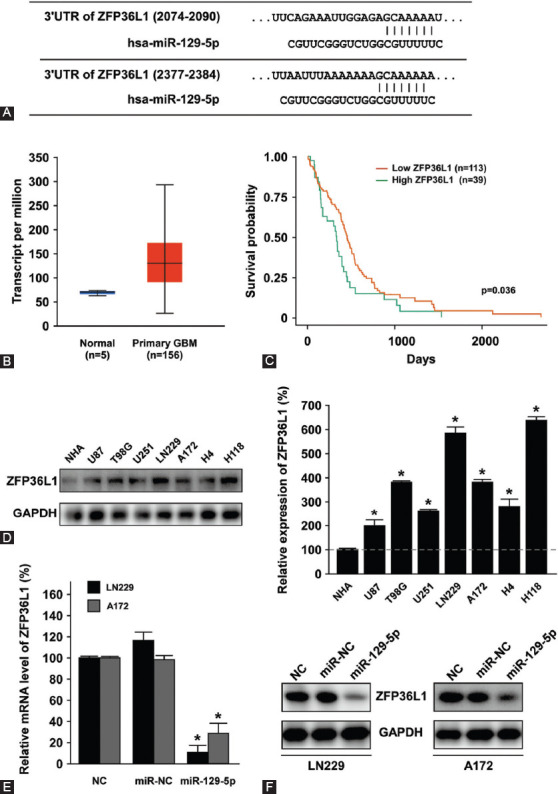
Relationship between miR-129-5p and its downstream target gene ZFP36L1. (A) Prediction of miR-129-5p binding sites in the 3’-UTRs of ZFP36L1 gene by TargetScan analysis. (B and C) Expression of ZFP36L1 and survival analysis in GBM patients using the UALCAN dataset. ZFP36L1 expression was upregulated in GBM tissues (n = 156) compared with normal brain tissues (n = 5). Higher ZFP36L1 expression was associated with poor survival. (D) ZFP36L1 expression was significantly upregulated in GBM cell lines LN229, A172, U87, T98G, U251, H4, and H118 compared with NHA. Differences were found to be statistically significant at *p < 0.05. (E and F) MiR-129-5p negatively regulated the expression of ZFP36L1 in LN229 and A172 cells. Overexpression of miR-129-5p in LN229 and A172 cells significantly suppressed the mRNA level (E) and protein level (F) of ZFP36L1. GAPDH was used as an internal control. Differences between miR-129-5p and miR-NC were found to be statistically significant at *p < 0.05. UTR: Untranslated region; ZFP36L1: ZFP36 ring finger protein-like 1; GBM: Glioblastoma multiforme; NHAs: Normal human astrocytes; GAPDH: Glyceraldehyde 3-phosphate dehydrogenase.

To further investigate the underlying mechanism of miR-129-5p-mediated inhibition of cell proliferation, migration and colony-forming ability in GBM, TargetScan bioinformatics database was used and ZFP36L1 was identified as a potential target gene of miR-129-5p (Figure 4A). UALCAN was used to examine the expression of ZFP36L1 in GBM samples. As shown in Figure 4B, the expression of ZFP36L1 in GBM samples (n = 156) was significantly higher than that in normal brain samples (n = 5) (*p* < 0.0005). In addition, survival analysis showed that patients with higher expression of ZFP36L1 had a significantly increased risk of death (Figure 4C).

Next, the expression of ZFP36L1 was analyzed in NHA and GBM cell lines. As shown in Figure 4D, ZFP36L1 was significantly upregulated to 2–6.5 fold in the seven GBM cell lines, suggesting that miR-129-5p was negatively correlated with ZFP36L1 in GBM. To further validate this hypothesis, miR-129-5p mimics was overexpressed in LN229 and A172 cells, and the expression of ZFP36L1 was determined by qRT-PCR. As shown in Figure 4E, the overexpression of miR-129-5p significantly inhibited ZFP36L1 mRNA expression in both LN229 and A172 cells (*p* < 0.05). The results of Western blot analysis (Figure 4F) further supported the hypothesis that miR-129-5p overexpression suppresses ZFP36L1 protein expression. Taken together, these results indicated that miR-129-5p negatively regulates the expression of ZFP36L1 in GBM.

### Overexpression of ZFP36L1 reverses the inhibitory effect of miR-129-5p in GBM

**FIGURE 5:**
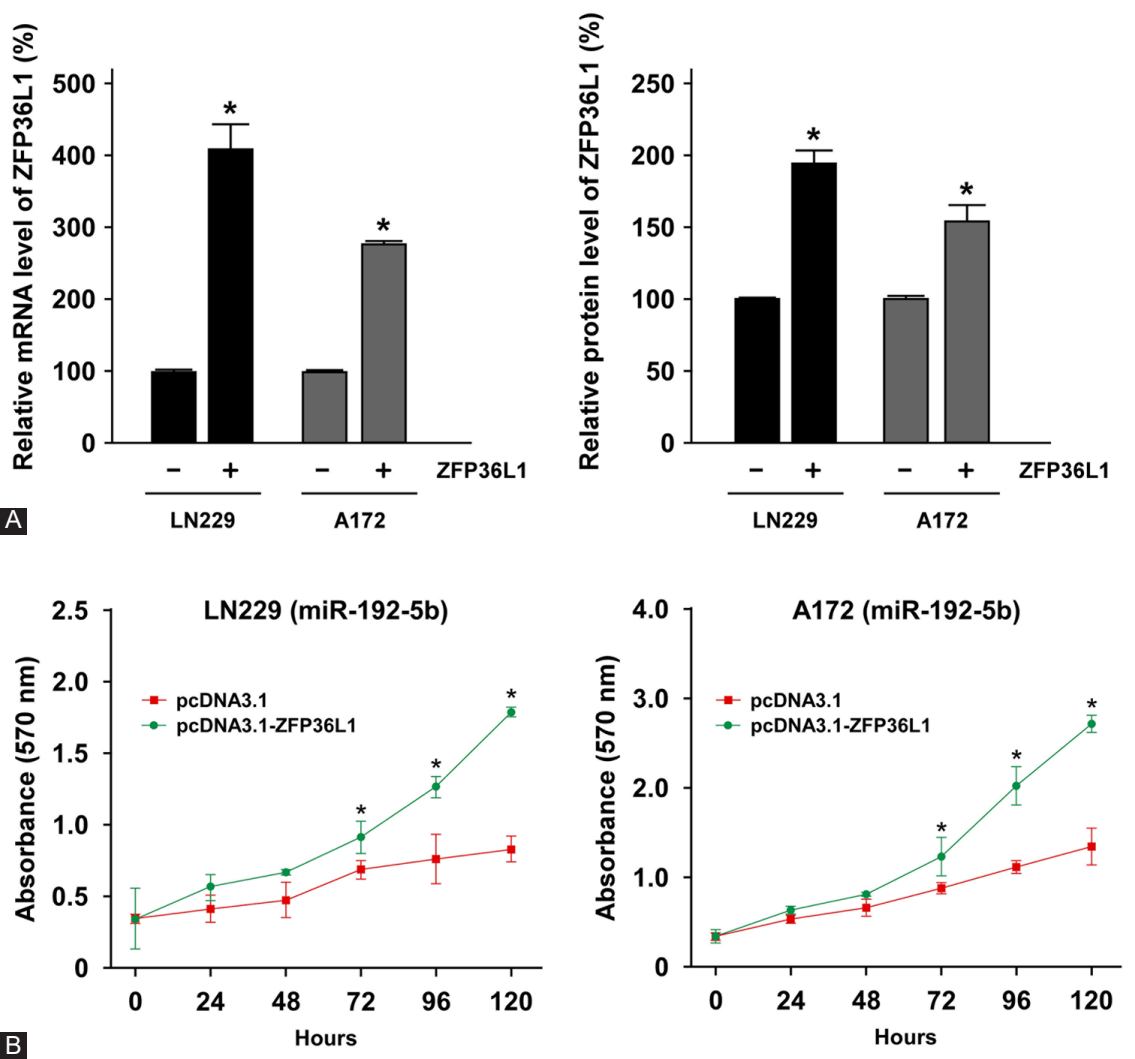
ZFP36L1 overexpression reversed miR-129-5p-mediated proliferation inhibition in GBM. (A) MiR-129-5p-overexpressing LN229 and A172 cells were transfected with pcDNA3.1/+ vector or pcDNA3.1/ZFP36L1, and the expression of ZFP36L1 was determined by quantitative reverse transcription PCR and Western blotting analysis. The mRNA (left panel) and protein (right panel) levels of ZFP36L1 were significantly increased after the transfection. (B) Following the transfection, the growth rates of LN229 and A178 cells were determined by CCK-8 assay. The absorbance was recorded at 450 nm for every 24 h. ZFP36L1 overexpression restored the proliferation inhibition by miR-129-5p in LN229 and A178 cells. ZFP36L1: ZFP36 ring finger protein-like 1; GBM: glioblastoma multiforme. Differences were considered statistically significant at *p < 0.05.

Next, we examined whether miR-129-5p-regulated ZFP36L1 expression is involved in GBM proliferation, migration, and colony forming ability. LN229 and A172 cells with overexpressed miR-129-5p were transfected with pcDNA3.1-ZFP36L1 to increase the level of ZFP36L1, and the cells in the control group were transfected with pcDNA3.1 vector. The expression of ZFP36L1 was confirmed by qRT-PCR and Western blot (Figure 5A). Compared to pcDNA3.1 control group, transfection with pcDNA3.1-ZFP36L1 successfully increased the mRNA and protein levels of ZFP36L1 in both LN229 and A172 cells (*p* < 0.05). Afterwards, we examined whether miR-129-5p-mediated inhibition of GBM cell proliferation could be reversed by increased expression of ZFP36L1. As shown in Figure 5B, ZFP36L1 overexpression significantly restored miR-129-5p-mediated proliferation inhibition in LN229 and A172 cells (*p* < 0.05), suggesting that ZFP36L1 plays a crucial role in GBM proliferation.

**FIGURE 6:**
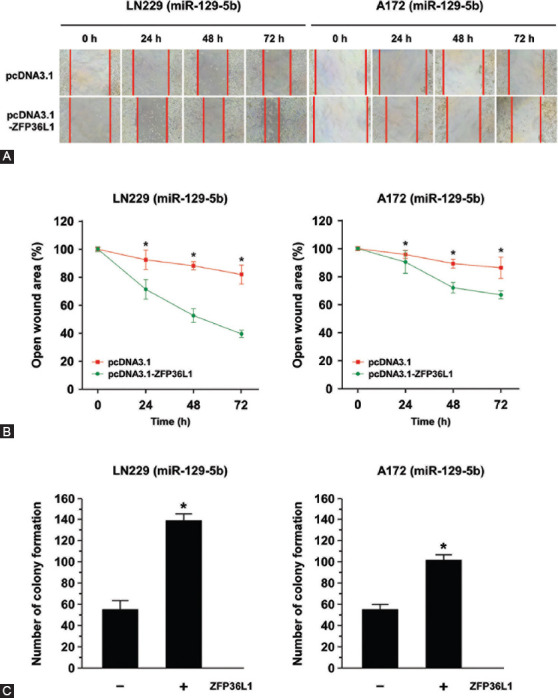
ZFP36L1 overexpression reversed miR-129-5p-mediated migration inhibition and suppression of colony-forming ability in GBM. (A) Photographs of artificially induced wounds in the monolayers of miR-129-5p-expressing LN229 and A172 cells with/without ZFP36L1 overexpression at different time points. ZFP36L1 overexpression increased LN229 and A172 cell migration ability. (B) Compared to the empty-vector transfected cells, the open wound area in pcDNA3.1-ZFP36L1 transfected LN229 and A172 cells were significantly reduced at 24, 48, and 72 h of culture. The wound-healing results were expressed as the percentage of migration ability at 0 h (100%). Data are presented as the means of triplicate experiments. Differences were found to be statistically significant at *p < 0.05. (C) After the transfection with ZFP36L1, the colony formation efficiency of LN229 and A172 cells was determined by counting the number of colonies formed. Overexpression of ZFP36L1 increased the numbers of colony formation. Differences were found to be statistically significant at *p < 0.05. GBM: Glioblastoma multiforme; ZFP36L1: ZFP36 ring finger protein-like 1.

We next examined the role of ZFP36L1 in GBM cell migration, invasion, and colony forming ability. The results of wound-healing assay showed that ZFP36L1 overexpression increased tumor migration ability (Figure 6A). Compared to empty-vector transfected cells, the open wound area in pcDNA3.1-ZFP36L1 transfected cells was significantly reduced at 24, 48, and 72 h of culture in LN229 and A172 cells (*p* < 0.05, Figure 6B). Furthermore, miR-129-5p-mediated inhibition of colony-forming ability was restored by ZFP36L1 overexpression (Figure 6C).

Collectively, these experiments indicated that miRNA -129-5p negatively regulates ZFP36L1 expression to affect the proliferation, migration, and colony-forming abilities of LN229 and A172 GBM cells.

## DISCUSSION

Despite multiple treatment modalities are available for GBM, GBM remains the most common primary malignant brain tumor, with a poor prognosis. In the past few years, increasing attention has been paid to the role of microRNAs in the development, diagnosis, and prognosis of gliomas. In this study, we demonstrated that miR-129-5p and miR-129-5p-regulated target gene *ZFP36L1* are involved in GBM. We found that miR-129-5p was markedly downregulated in GBM tissues and seven GBM cell lines. Overexpression of miR-129-5p suppressed GBM cell proliferation, migration, and colony-forming ability. Furthermore, we identified that *ZFP36L1* was the downstream target of miR-129-5p and was responsible for miR-129-5p-mediated tumor proliferation, migration, and colony-forming ability. Overexpression of *ZFP36L1* in miR-129-5p-overexpressing GBM cells successfully restored all the features of GBM tumorigenesis. Overall, our study suggests that during the development of GBM, miR-129-5p expression is reduced and leads to higher expression of *ZFP36L1*, which results in increased proliferation, migration, and colony forming ability of GBM cells.

MiR-129-5p has recently received considerable attention as a tumor suppressor. In gastric cancer, miR-129-5p was found to attenuate tumor progression by targeting high mobility group box 1 (HMGB1) [27], WW domain containing E3 ubiquitin protein ligase 1 (WWP1) [28], disintegrin and metalloproteinase domain-containing protein 9 (ADAM9) [46], solute carrier family 2 member 3 (SLC2A3) [47], collagen, type I, alpha 1 (COLA1) [48], and interleukin (IL)-8 [49]. In addition, miR-129-5p was shown to regulate cell invasion and metastasis in lung cancer [32], hepatocellular carcinoma [50], pancreatic [29], and prostate cancer [31]. Gao *et al*. found that the expression of miR-129-5p was significantly decreased in human prostate cancer tissues, and downregulation of miR-129-5p increased ETS variant transcription factor 1 (ETV1) expression. Overexpression of ETV1 reversed miR-129-5p mimics inducing cell growth arrest [31]. On the other hand, Gu *et al*. found that DNA (cytosine-5)-methyltransferase 3A (DNMT3A), a tumor suppressor in glioma, was negatively regulated by miR-129-5p [51]. Overexpression of DNMT3A partially attenuates miR-129-5p-inhibited cell proliferation, suggesting that other unidentified downstream targets of miR-129-5p may affect GBM proliferation as well as other features of GBM tumorigenesis, such as migration and colony-forming ability.

In this study, miR-129-5p was found to be significantly downregulated in LN229, A172, U87, T98G, U251, H4, and LN118 cells and in GBM tissue samples. To further explore the specific biological role of miR-129-5p in GBM, the two cell lines with the lowest expression of miR-129-5p, LN229 and A172, were selected to restore the expression of miR-129-5p. Notably, miR-129-5p overexpression not only inhibited cell proliferation and migration, but also suppressed the colony-forming ability. These results indicated that miR-129-5p can be considered as a tumor suppressor during GBM development and that miR-129-5p may be used as a potential diagnostic and prognostic biomarker in GBM patients.

In addition to its association with tumorigenesis, miR-129-5p was implicated in other diseases. A study by Yang *et al*. suggested that dysregulation of miR-129-5p might contribute to the development of intervertebral disc degeneration by targeting bone morphogenetic protein 2 (BMP2) expression [25]. In patients with intervertebral disc degeneration, miR-129-5p was downregulated along with BMP2 upregulation. Overexpression of miR-129-5p apparently increased the survival of neural progenitor cells, whereas downregulation of miR-129-5p increased apoptosis. Moreover, restored BMP2 expression reversed the effect of miR-129-5p. On the other hand, miR-129-5p was also recently suggested to play a crucial role in the protection of cardiac function [52]. In a chronic heart failure rat model, miR-129-5p transfection not only improved heart function and hemodynamic parameters, but also attenuated oxidative stress and inflammation factors. In addition, a study by Zeng *et al*. demonstrated that miR-129-5p may be a potential candidate for the treatment of Alzheimer’s disease [23]. Overexpression of miR-129-5p promoted neuronal cell proliferation, suppressed apoptosis, and decreased the inflammatory reaction. These studies suggest that miR-129-5p is involved in different biological processes, which deserves further investigation.

It is difficult to develop efficient treatment against GBM, because GBM is characterized by many dysregulated pathways. Currently, maximal surgical resection combined with postoperative radiotherapy and chemotherapy are the standard treatment of GBM. However, chemoresistance is the typical hallmark of recurrent GBM. More than 90% of GBM relapsed at the primary tumor site, and 5% of GBM occurred as multiple lesions after treatment [53]. Ma *et al*. demonstrated that miR-129-5p was able to inhibit the stemness and chemoresistance of non-small cell lung cancer (NSCLC) cells [54]. Namely, overexpression of miR-129-5p suppressed the ability of sphere formation and the expression of stemness makers, including octamer-binding transcription factor 4 (Oct4), SRY-box 2 (Sox2), and Nanog in NSCLC cells [54]. Furthermore, miR-129-5p/HMGB1 axis [55] and NONHSAT101069/miR-129-5p axis [56] were found to regulate the radiosensitivity and epirubicin resistance of breast cancer cells, respectively. In this study, colony-forming assay was conducted to indirectly investigate the stemness ability of GBM cells. Remarkably, our results showed that miR-129-5p significantly suppressed the colony-forming ability of LN229 and A172 GBM cells. This implied that miR-129-5p may affect GBM stemness and warrants further investigation in the future.

The RNA-binding protein ZFP36L1 is able to directly bind to the 3’-UTRs of their target mRNAs and promote mRNA decay. However, a few studies focused on the role of ZFP36L1 in tumorigenesis. Loh *et al*. suggested that ZFP36L1 may function as a tumor suppressor [57]. Forced expression of ZFP36L1 inhibited cell proliferation in bladder and breast cancer cells. On the other hand, a study by Zindy *et al*. demonstrated that ZFP36L1 was upregulated in hepatocellular carcinoma, implying that ZFP36L1 may be oncogenic in liver cancer [58]. In this study, we found that ZFP36L1 was highly expressed in GBM cell lines and tissues samples. Remarkably, GBM patients with higher expression of ZFP36L1 were associated with poor survival. In contrast to the study of Loh *et al*. on bladder and breast cancer cells [57], ZFP36L1 overexpression in GBM cells not only enhanced cell proliferation, but also increased cell migration and colony-forming ability. This suggests that ZFP36L1 may play different biological roles in different tumors and be a potential target for GBM treatment. This deserves further investigation.

The limitation of this study was the small number of GBM patients available in the databases. Although the current study found that miR-129-5p and ZFP36L1 are involved in GBM tumorigenesis, it remains unclear whether the expression of miR-129-5p and ZFP36L1 are associated with the overall survival, disease-free survival, and recurrence-free survival of GBM patients. Thus, further prospective studies should be conducted to investigate the association of miR-129-5p and ZFP36L1 with patient survival taking into consideration important prognostic confounders.

## CONCLUSION

The present findings revealed a tumor suppressor role of miR-129-5p/ZFP36L1 axis in GBM cell proliferation, migration, and colony-forming ability. Importantly, ZFP36L1 expression in GBM was negatively regulated by miR-129-5p and associated with poor survival. Altogether, our study not only identified a new tumor suppressor role of miR-129-5p/ZFP36L1 axis in GBM tumorigenesis, but also provided potential targets for the treatment of GBM patients.

## References

[1] Ostrom QT, Gittleman H, Truitt G, Boscia A, Kruchko C, Barnholtz-Sloan JS (2018). CBTRUS statistical report:Primary brain and other central nervous system tumors diagnosed in the United States in 2011-2015. Neuro Oncol.

[2] Batash R, Asna N, Schaffer P, Francis N, Schaffer M (2017). Glioblastoma multiforme, diagnosis and treatment;recent literature review. Curr Med Chem.

[3] Lukas RV, Wainwright DA, Ladomersky E, Sachdev S, Sonabend AM, Stupp R (2019). Newly diagnosed glioblastoma:A review on clinical management. Oncology (Williston Park).

[4] Stoyanov GS, Dzhenkov D, Ghenev P, Iliev B, Enchev Y, Tonchev AB (2018). Cell biology of glioblastoma multiforme:From basic science to diagnosis and treatment. Med Oncol.

[5] Bartel DP (2009). MicroRNAs:Target recognition and regulatory functions. Cell.

[6] Kim M, Civin CI, Kingsbury TJ (2019). MicroRNAs as regulators and effectors of hematopoietic transcription factors. Wiley Interdiscip Rev RNA.

[7] Mendell JT, Olson EN (2012). MicroRNAs in stress signaling and human disease. Cell.

[8] Mitchell PS, Parkin RK, Kroh EM, Fritz BR, Wyman SK, Pogosova-Agadjanyan EL (2008). Circulating microRNAs as stable blood-based markers for cancer detection. Proc Natl Acad Sci U S A.

[9] Kawaguchi T, Yan L, Qi Q, Peng X, Gabriel EM, Young J (2017). Overexpression of suppressive microRNAs, miR-30a and miR-200c are associated with improved survival of breast cancer patients. Sci Rep.

[10] Zhou X, Tao H (2018). Overexpression of microRNA-936 suppresses non-small cell lung cancer cell proliferation and invasion via targeting E2F2. Exp Ther Med.

[11] Zhao G, Wang B, Liu Y, Zhang JG, Deng SC, Qin Q (2013). miRNA-141, downregulated in pancreatic cancer, inhibits cell proliferation and invasion by directly targeting MAP4K4. Mol Cancer Ther.

[12] Bao L, Zhang M, Han S, Zhan Y, Guo W, Teng F (2018). MicroRNA-500a promotes the progression of hepatocellular carcinoma by post-transcriptionally targeting BID. Cell Physiol Biochem.

[13] Zhi T, Jiang K, Zhang C, Xu X, Wu W, Nie E (2017). MicroRNA-1301 inhibits proliferation of human glioma cells by directly targeting N-Ras. Am J Cancer Res.

[14] Xu X, Cai N, Zhi T, Bao Z, Wang D, Liu Y (2017). MicroRNA-1179 inhibits glioblastoma cell proliferation and cell cycle progression via directly targeting E2F transcription factor 5. Am J Cancer Res.

[15] Wang D, Zhi T, Xu X, Bao Z, Fan L, Li Z (2017). MicroRNA-936 induces cell cycle arrest and inhibits glioma cell proliferation by targeting CKS1. Am J Cancer Res.

[16] Zhi T, Jiang K, Xu X, Yu T, Wu W, Nie E (2017). MicroRNA-520d-5p inhibits human glioma cell proliferation and induces cell cycle arrest by directly targeting PTTG1. Am J Transl Res.

[17] Wu W, Zhou X, Yu T, Bao Z, Zhi T, Jiang K (2017). The malignancy of miR-18a in human glioblastoma via directly targeting CBX7. Am J Cancer Res.

[18] Xu TJ, Qiu P, Zhang YB, Yu SY, Xu GM, Yang W (2019). MiR-148a inhibits the proliferation and migration of glioblastoma by targeting ITGA9. Hum Cell.

[19] Guo XB, Zhang XC, Chen P, Ma LM, Shen ZQ (2019). miR378a3p inhibits cellular proliferation and migration in glioblastoma multiforme by targeting tetraspanin 17. Oncol Rep.

[20] Qian C, Wang B, Zou Y, Zhang Y, Hu X, Sun W (2019). MicroRNA 145 enhances chemosensitivity of glioblastoma stem cells to demethoxycurcumin. Cancer Manag Res.

[21] Yang Y, Dodbele S, Park T, Glass R, Bhat K, Sulman EP (2019). MicroRNA-29a inhibits glioblastoma stem cells and tumor growth by regulating the PDGF pathway. J Neurooncol.

[22] Hua L, Zhu G, Wei J (2018). MicroRNA-1 overexpression increases chemosensitivity of non-small cell lung cancer cells by inhibiting autophagy related 3-mediated autophagy. Cell Biol Int.

[23] Zeng Z, Liu Y, Zheng W, Liu L, Yin H, Zhang S (2019). MicroRNA-129-5p alleviates nerve injury and inflammatory response of Alzheimer's disease via downregulating SOX6. Cell Cycle.

[24] Valenti MT, Deiana M, Cheri S, Dotta M, Zamboni F, Gabbiani D (2019). Physical exercise modulates miR-21-5p, miR-129-5p, miR-378-5p, and miR-188-5p expression in progenitor cells promoting osteogenesis. Cells.

[25] Yang W, Sun P (2019). Downregulation of microRNA-129-5p increases the risk of intervertebral disc degeneration by promoting the apoptosis of nucleus pulposus cells via targeting BMP2. J Cell Biochem.

[26] Yang W, Pan Y, Guan P, Li X, You C (2019). Bioinformatics analysis of COL1A1 regulated by miR-129-5p as a potential therapeutic target for gastric cancer [Article in Chinese]. Nan Fang Yi Ke Da Xue Xue Bao.

[27] Wang S, Chen Y, Yu X, Lu Y, Wang H, Wu F (2019). miR-129-5p attenuates cell proliferation and epithelial mesenchymal transition via HMGB1 in gastric cancer. Pathol Res Pract.

[28] Ma L, Chen X, Li C, Cheng R, Gao Z, Meng X (2019). miR-129-5p and -3p co-target WWP1 to suppress gastric cancer proliferation and migration. J Cell Biochem.

[29] Qiu Z, Wang X, Shi Y, Da M (2019). miR-129-5p suppresses proliferation, migration, and induces apoptosis in pancreatic cancer cells by targeting PBX3. Acta Biochim Biophys Sin (Shanghai).

[30] Li J, Sun S, Chen W, Yuan K (2019). Small nucleolar RNA host gene 12 (SNHG12) promotes proliferation and invasion of laryngeal cancer cells via sponging miR-129-5p and potentiating WW domain-containing E3 ubiquitin protein ligase 1 (WWP1) expression. Med Sci Monit.

[31] Gao G, Xiu D, Yang B, Sun D, Wei X, Ding Y (2019). miR-129-5p inhibits prostate cancer proliferation via targeting ETV1. Onco Targets Ther.

[32] Li G, Xie J, Wang J (2019). Tumor suppressor function of miR-129-5p in lung cancer. Oncol Lett.

[33] Shaker OG, Abdelwahed MY, Ahmed NA, Hassan EA, Ahmed TI, Abousarie MA (2019). Evaluation of serum long noncoding RNA NEAT and miR-129-5p in hepatocellular carcinoma. IUBMB Life.

[34] Zeng A, Yin J, Li Y, Li R, Wang Z, Zhou X (2018). miR-129-5p targets Wnt5a to block PKC/ERK/NF-kappaB and JNK pathways in glioblastoma. Cell Death Dis.

[35] Blackshear PJ, Perera L (2014). Phylogenetic distribution and evolution of the linked RNA-binding and NOT1-binding domains in the tristetraprolin family of tandem CCCH zinc finger proteins. J Interferon Cytokine Res.

[36] Gupta G, Bebawy M, Pinto TJA, Chellappan DK, Mishra A, Dua K (2018). Role of the tristetraprolin (zinc finger protein 36 homolog) gene in cancer. Crit Rev Eukaryot Gene Expr.

[37] Tarling EJ, Clifford BL, Cheng J, Morand P, Cheng A, Lester E (2017). RNA-binding protein ZFP36L1 maintains posttranscriptional regulation of bile acid metabolism. J Clin Invest.

[38] Bye AJH, Pugazhendhi D, Woodhouse S, Brien P, Watson R, Turner M (2018). The RNA-binding proteins Zfp36l1 and Zfp36l2 act redundantly in myogenesis. Skelet Muscle.

[39] Galloway A, Saveliev A, Lukasiak S, Hodson DJ, Bolland D, Balmanno K (2016). RNA-binding proteins ZFP36L1 and ZFP36L2 promote cell quiescence. Science.

[40] Hodson DJ, Janas ML, Galloway A, Bell SE, Andrews S, Li CM (2010). Deletion of the RNA-binding proteins ZFP36L1 and ZFP36L2 leads to perturbed thymic development and T lymphoblastic leukemia. Nat Immunol.

[41] Stumpo DJ, Byrd NA, Phillips RS, Ghosh S, Maronpot RR, Castranio T (2004). Chorioallantoic fusion defects and embryonic lethality resulting from disruption of Zfp36L1, a gene encoding a CCCH tandem zinc finger protein of the Tristetraprolin family. Mol Cell Biol.

[42] Planel S, Salomon A, Jalinot P, Feige JJ, Cherradi N (2010). A novel concept in antiangiogenic and antitumoral therapy:Multitarget destabilization of short-lived mRNAs by the zinc finger protein ZFP36L1. Oncogene.

[43] Suk FM, Chang CC, Lin RJ, Lin SY, Liu SC, Jau CF (2018). ZFP36L1 and ZFP36L2 inhibit cell proliferation in a cyclin D-dependent and p53-independent manner. Sci Rep.

[44] Huang CC, Chang WS (2009). Cooperation between NRF-2 and YY-1 transcription factors is essential for triggering the expression of the PREPL-C2ORF34 bidirectional gene pair. BMC Mol Biol.

[45] Huang CC, Chen KL, Cheung CHA, Chang JY (2013). Autophagy induced by cathepsin S inhibition induces early ROS production, oxidative DNA damage, and cell death via xanthine oxidase. Free Radic Biol Med.

[46] Liu Q, Jiang J, Fu Y, Liu T, Yu Y, Zhang X (2018). MiR-129-5p functions as a tumor suppressor in gastric cancer progression through targeting ADAM9. Biomed Pharmacother.

[47] Chen D, Wang H, Chen J, Li Z, Li S, Hu Z (2018). MicroRNA-129-5p regulates glycolysis and cell proliferation by targeting the glucose transporter SLC2A3 in gastric cancer cells. Front Pharmacol.

[48] Wang Q, Yu J (2018). MiR-129-5p suppresses gastric cancer cell invasion and proliferation by inhibiting COL1A1. Biochem Cell Biol.

[49] Jiang Z, Wang H, Li Y, Hou Z, Ma N, Chen W (2016). MiR-129-5p is down-regulated and involved in migration and invasion of gastric cancer cells by targeting interleukin-8. Neoplasma.

[50] Zhang D, Cao J, Zhong Q, Zeng L, Cai C, Lei L (2017). Long noncoding RNA PCAT-1 promotes invasion and metastasis via the miR-129-5p-HMGB1 signaling pathway in hepatocellular carcinoma. Biomed Pharmacother.

[51] Gu X, Gong H, Shen L, Gu Q (2018). MicroRNA-129-5p inhibits human glioma cell proliferation and induces cell cycle arrest by directly targeting DNMT3A. Am J Transl Res.

[52] Xiao N, Zhang J, Chen C, Wan Y, Wang N, Yang J (2019). miR-129-5p improves cardiac function in rats with chronic heart failure through targeting HMGB1. Mamm Genome.

[53] Loeffler JS, Alexander E, Hochberg FH, Wen PY, Morris JH, Schoene WC (1990). Clinical patterns of failure following stereotactic interstitial irradiation for malignant gliomas. Int J Radiat Oncol Biol Phys.

[54] Ma Z, Cai H, Zhang Y, Chang L, Cui Y (2017). MiR-129-5p inhibits non-small cell lung cancer cell stemness and chemoresistance through targeting DLK1. Biochem Biophys Res Commun.

[55] Luo J, Chen J, He L (2015). Mir-129-5p attenuates irradiation-induced autophagy and decreases radioresistance of breast cancer cells by targeting HMGB1. Med Sci Monit.

[56] Yao N, Fu Y, Chen L, Liu Z, He J, Zhu Y (2019). Long non-coding RNA NONHSAT101069 promotes epirubicin resistance, migration, and invasion of breast cancer cells through NONHSAT101069/miR-129-5p/Twist1 axis. Oncogene.

[57] Loh XY, Sun QY, Ding LW, Mayakonda A, Venkatachalam N, Yeo MS (2020). RNA-binding protein ZFP36L1 suppresses hypoxia and cell cycle signaling. Cancer Res.

[58] Zindy PJ, L'Helgoualc'h A, Bonnier D, Le Bechec A, Bourd-Boitin K, Zhang CX (2006). Upregulation of the tumor suppressor gene menin in hepatocellular carcinomas and its significance in fibrogenesis. Hepatology.

